# Development and Evaluation of Up-Converting Phosphor Technology-Based Lateral Flow Assay for Quantitative Detection of NT-proBNP in Blood

**DOI:** 10.1371/journal.pone.0171376

**Published:** 2017-02-02

**Authors:** Xiaoli Yang, Liping Liu, Qingfang Hao, Deyong Zou, Xiaoli Zhang, Liping Zhang, Hongmei Li, Yong Qiao, Huansheng Zhao, Lei Zhou

**Affiliations:** 1 Clinical laboratory, General Hospital of Chinese People’s Armed Police Forces, Hai Dian District, Beijing, China; 2 Beijing Hotgen Biotech Co. Ltd., Da Xing Industrial Development Zone, Beijing, China; 3 Laboratory of Analytical Microbiology, State Key Laboratory of Pathogen and Biosecurity, Beijing Institute of Microbiology and Epidemiology, Beijing, P.R. China; 4 Beijing Key Laboratory of POCT for Bioemergency and Clinic (No. BZ0329), Beijing, P.R. China; Institut d'Investigacions Biomediques de Barcelona, SPAIN

## Abstract

A newly assay, up-converting phosphor technology-based lateral flow (UPT-LF) assay, was developed for rapid and quantitative detection of N-terminal fragment of B-type natriuretic peptide precursor (NT-proBNP), one of the most important serum molecular maker of heat failure, in plasma samples as a point of care testing (POCT) method for diagnosis of acute heart failure. Human plasma from 197 patients with acute heart failure and 200 healthy controls was assessed using the UPT-LF assay, in a comparison with a Roche Elecsys assay. The limit of detection of the UPT-LF assay, with a coefficient of variation (CV) of less than 15%, was 116 ng/L, which is lower than the clinical diagnosis cutoff (150 ng/mL). The linear range was 50–35,000 ng/L. The CVs were less than 10% for both UPT-LF and Roche Elecsys assays for plasma samples under different storages, demonstrating the good stability and reproducibility. There are certain linear correlations between the results of UPT-LF and Roche Elecsys assay for EDTA-K2 and heparin-anticoagulated plasma, as well as for serum samples. For UPT-LF assay, there is a significant correlation between the values derived from analysis of EDTA-K2 and heparin-anticoagulated plasma samples (R = 0.995). No statistically significant difference was found between serum and plasma samples for UPT-LF assay. Our results demonstrate that NT-proBNP levels in healthy adults are elevated with age and had a relationship with sex, and with the age increase the NT-proBNP levels of females are significantly higher than those of males (*p*<0.01). The UPT-LF assay has a high reproducibility, stability, sensitivity, specificity, and is consistent with Roche Elecsys assay, and therefore it could be used as a POCT method for the quantitative detection of NT-proBNP in blood for clinical diagnosis and research of acute heart failure.

## Introduction

Heart failure, caused by cardiac dysfunction and clinical syndromes, is a major health problem with high rates of mortality. Although deterioration of heart function can be delayed by drug therapy, it is not currently possible to cure heart failure. Patients appear to have a better prognosis when treated earlier. B-type natriuretic peptide (BNP) and N-terminal fragment of BNP precursor (NT-proBNP) are important biomarkers for the prediction and diagnosis of heart failure, with high specificity and sensitivity [1]. The increased BNP and NT-proBNP level was associated with underlying cardiac involvement [2] and mortality [3], especially be strongly recommended to identify heart failure for elderly [4], patient with stable angina [2] and left ventricular dysfunction [5], and pediatric patient with sepsis [6]. BNP is a member of the natriuretic factor family and is secreted by myocardial cells. BNP precursor (pro-BNP) is first synthesized with 108 amino acids and then is cut into activated BNP (32 amino acids) and inactivated NT-proBNP (76 amino acids). The ratio of NT-proBNP/BNP that is released into circulating blood in healthy individuals is 1.9–3.0:1, while it increases to 3.0–9.2:1 in cardiac patients. Moreover, NT-proBNP is passively and slowly cleared from the circulation (with a half-life of 120 minutes versus 22 minutes for BNP). Therefore, the detection of NT-proBNP is more significant than BNP for diagnostic purposes because of the higher stability due to the longer half-life time. Numerous studies have shown that the level of NT-proBNP can offer a more accurate reflection of damaged heart function as other cardiac markers [1, 7, 8]. Riezebos et al. found that the serum levels of NT- ProBNP reflect the severity and extent of ischemia in patients who are admitted with acute coronary syndrome (ACS) without ST-elevation [9, 10]. The measurement of NT-proBNP upon admission improves the early risk stratification of patients with ACS, and the predictive value of NT-proBNP for the prognosis of ACS is stronger than the left ventricular ejection fraction value [11]. McDonagh et al. analyzed several factors in 3,051 cases, such as age, sex, hypertension, diabetes, and coronary heart disease, among others [12]. Their multiple regression results showed that NT-proBNP is an independent predictor of heart failure. A high level of NT-proBNP suggests severe heart failure and a poor prognosis [13]. A measurement of plasma NT-proBNP is highly beneficial for the prediction of heart failure in the early stages, and for guiding treatment for elderly and patient with underlying cardiac involvement [2–6]. Rapid and accurate detection of NT-proBNP may have important implications for disease diagnosis and prognosis, guiding drug usage, and reducing the risk of death.

At present, two methods are predominantly used to detect the concentration of NT-proBNP. One is laboratory testing based on large-scale precision instrument, such as electrochemical luminescence [14] and Dimension RxL NT-proBNP method [15] based on fully-auto-biochemistry-analysis instruments, which have high sensitivity and accuracy, require large and sophisticated apparatus, and produce results in at least 2 hours. The other method is rapid bedside measurement, namely point of care testing (POCT) such as the colloidal gold semi-quantitative colorimetric method, microfluidic immunoassay [16], immunological turbidimetric assay [17], and other novel rapid assay [18], and they are rapid but their sensitivity is relatively low. The alternation of the two methods, laboratory testing or POCT method, in clinics is relatively common, in a similar manner to blood glucose assays. Discrepant results may generated by different methods and affect the judgment of clinicians [19], therefore the consistency of the two methods is very important for correct diagnosis, prompt treatment and disease monitoring. As a whole, the establishment of a rapid and convenient POCT method with high sensitivity and consistency with laboratory testing is required, especially for acute heat failure.

Up-converting phosphor technology (UPT) was developed using up-converting phosphor particles (UCP) as reporter, a crystal materials synthesized from rare earth metal elements that can be excited using infrared light and emit visible light. Compared with fluorescein and fluorescent particles, it has a cleaner background and higher sensitivity and stability. By combining the UPT technology and immune chromatography, to form a UPT lateral flow (UPT-LF) assay, we can scan the luminescent particles on test strips and quality control strips to make a quantitative evaluation. To date, UPT has been used in the detection of bacteria [20–22], virus [23], parasites [24], and toxin [25,26], as well as detection of interferon [27] and nucleic acid [28–30], because of its sensitivity improvement, quantitative measurements, absence of autofluorescence, and robust performance for complex samples [21,22].

In the present study, we detect NT-proBNP using a UPT-LF assay, for the first time, and evaluate the performance of the assay to satisfy the requirements of POCT for acute heart failure. The results of UPT-LF assay were consistent with Roche Elecsys assay (Germany), an electrochemical luminescence assay with fully-auto-biochemistry-analysis instruments, and the high reproducibility, stability, sensitivity, specificity make UPT-LF assay suitable as a POCT method for quantitative detection of NT-proBNP in blood for clinical diagnosis and research.

## Results and Discussion

### UPT-LF assay for detection of NT-proBNP

**Fig 1:**
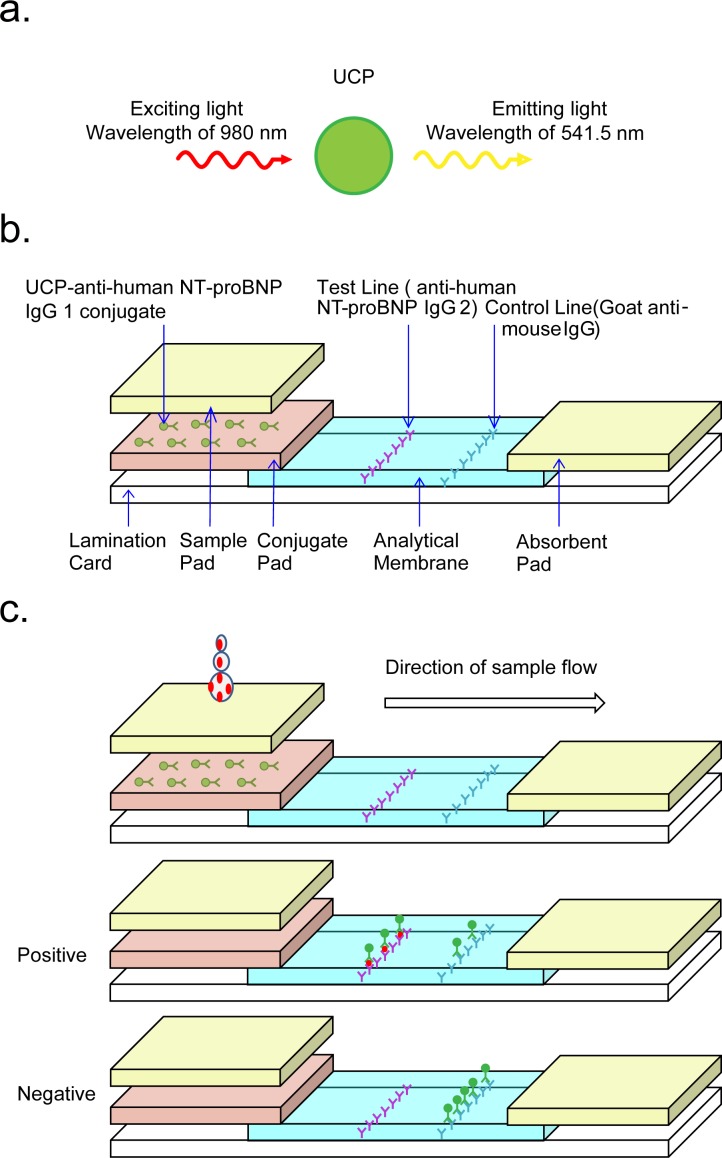
Configuration of the up-converting phosphor technology-based lateral flow strip. (a) The excitation and emission spectrum peaks of UCP were 980 nm and 541.5 nm, respectively. (b) The membrane of the UPT-LF strip consists of a test line (T line) and control line (C line), which have been coated with anti-human NT-proBNP monoclonal antibody and goat anti-mouse IgG respectively. The UCP particles were conjugated covalently with the other anti-human NT-proBNP antibody as a reporter. (c) For sample containing NT-proBNP, UCP reporters were captured on both the T line and C line, while they were only captured on the C line for sample without NT-proBNP. The signal from the UCP reporters indicated the strength of the immune reaction.

UPT-LF strips were prepared for the detection of NT-proBNP. As shown in Fig 1, the principle is as follows: the analytical membranes of the UPT-LF strips were functionalized with anti-human NT-proBNP (monoclonal antibody) and goat anti-mouse IgG as a test line and control line (T line and C line), respectively. The conjugate pad was provided with the conjugate of UCP particles and another anti-human NT-proBNP IgG. For positive samples (NT-proBNP containing samples), UCP reporters were captured on both the T line and the C line. However, UCP reporters were only captured on the C line when the negative samples (samples not containing any NT-proBNP) were analyzed. The signal from the UCP reporters indicated the strength of the immune reaction. The ratios of signal values on T line and C line (T/C ratio) obtained by the strip reader of UPT biosensor [31, 32] were compared with a standard curve. Finally, the concentrations of NT-proBNP were calculated and could be read directly on the screen.

### Linearity and sensitivity of the UPT-LF assay

**Fig 2:**
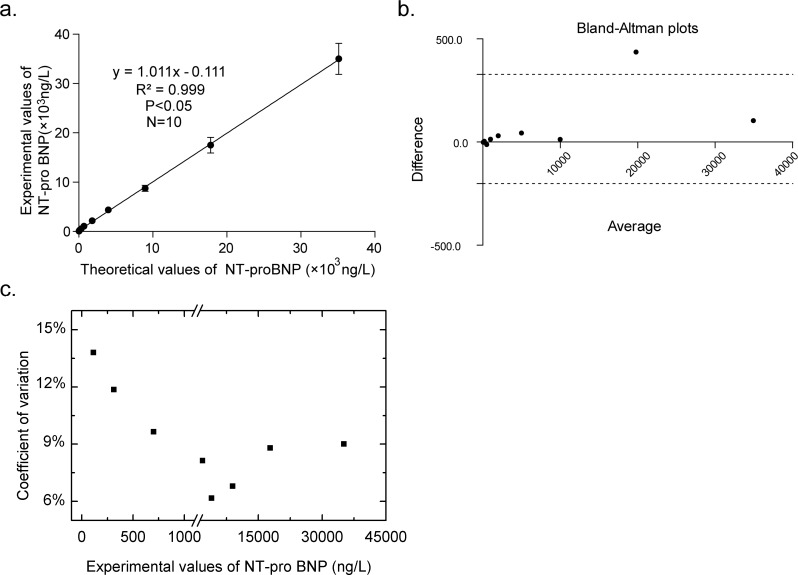
Linearity and sensitivity of the UPT-LF assay. (a) The linear scope of NT-proBNP detected by the UPT-LF assay reached 50–35000 ng/L. (b) Bland–Altman plots for linearity of the UPT-LF assay, the dotted lines represent the confidence interval for 95% limits of agreement. (c) The coefficient of variation values for different concentrations of NT-proBNP above clinical diagnostic cutoff (150 ng/L) were all less than 15%.

The precision, stability and linearity of the UPT-LF assay were evaluated according to the America Clinical And Laboratory Standards Institute (CLSI) standards. According to the results of the linear analysis, the UPT-LF assay did not show a linear relationship when the concentration of NT-proBNP was more than 35,000 ng/L (data not shown). Therefore, we chose ten concentrations within 50–35,000 ng/L, which showed a significant linear relationship. As shown in Fig 2A, the equation is Y = 1.011 X − 0.111, (R^2^ = 0.999, *p* < 0.05, n = 10), showing a high precision. Bland–Altman plots were used to evaluate the consistency between the UPT-LF assay and the Roche Elecsys assay (Fig 2B). The bias was 15.9% and the correlation coefficient (r) was 0.9997 (*p*<0.0001).

The clinical diagnostic cutoff for NT-proBNP is 150 ng/L, while the coefficient of variation (CV) of the UPT-LF assay for 116 ng/L was less than 15%, and less than 10% for above 700 ng/mL, demonstrating a good sensitivity and accuracy for clinical applications.

### Stability and reproducibility of the UPT-LF assay

**Fig 3:**
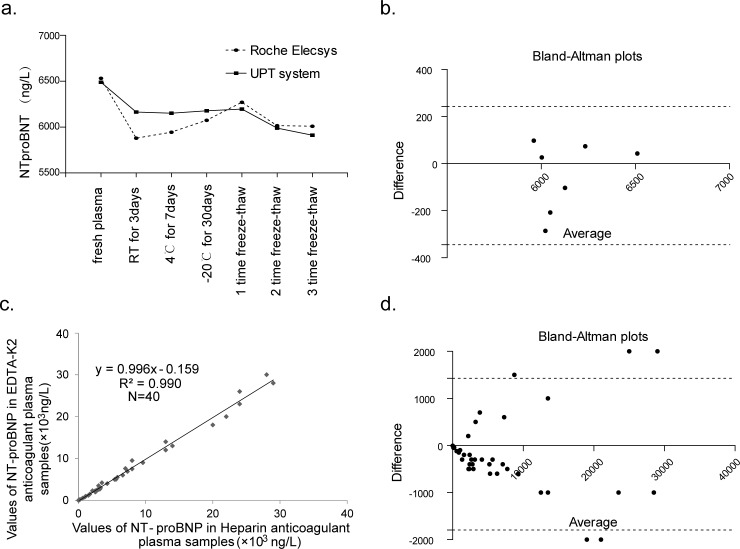
Stability of the UPT-LF assay. The coefficient of variation for the detection results of the UPT-LF assay and the Roche Elecsys assay were all less than 10% under different sample storage conditions (a), and the bias between the two methods (b) were -0.8%, demonstrating the good stability and reproducibility of UPT-LF assay as Roche Elecsys assay. The significant correlation between the values derived form the EDTA-K2- and 40 heparin-anticoagulated plasma specimens (c) and the bias of 3.84% (d) proved that UPT-LF assay were scarcely affected by anticoagulants. the dotted lines represent the 95% limits of agreement.

The stability and reproducibility of the UPT-LF assay was examined across a number of different circumstances. As shown in Fig 3A, the influence of sample storage temperature on the UPT-LF assay was less than with the Roche Elecsys assay. Compared with the fresh plasma, the coefficients of variation were 5.0%, 5.2%, 4.8% for the plasma stored at room temperature for 3 days, 4°C for 7 days, and −20°C for 30 days, as assessed using the UPT-LF assay, while those obtained when using the Roche Elecsys assay were 10%, 9%, and 7%, respectively (*p* = 0.0456). The level of the detected analyte was reduced with the increase in the number of freezing and thawing cycles for both methods, and CVs were less than 10% even after three freeze/thaw cycles (CVs of UPT-LF assay for one, two, three time freeze-thaw is 4.5%, 7.7%, 8.9%, while that of Roche Elecsys assay is 4.0%, 7.9% and 8.0% respectively). The results of the Bland–Altman bias analysis showed a bias of −0.8% between the two methods (Fig 3B), indicating that the stability and reproducibility of UPT-LF assay are as good as the Roche Elecsys assay.

To explore the effects of anticoagulants on the results [33], we evaluated 40 EDTA-K2- and 40 heparin-anticoagulated plasma specimens using the UPT-LF assay. There is a significant correlation (R = 0.995, *p* < 0.01) between the values derived from analysis of both types of anticoagulated sample (Fig 3C). A Cusum test on the regression linear showed no statistical significance (*p* > 0.05), and the result of the Bland–Altman bias analysis showed a bias of 3.84% between the two anticoagulants (Fig 3D), proving the anticoagulants have little influence on UPT-LF assay.

### The correlation between the UPT-LF and Roche Elecsys assays

**Fig 4:**
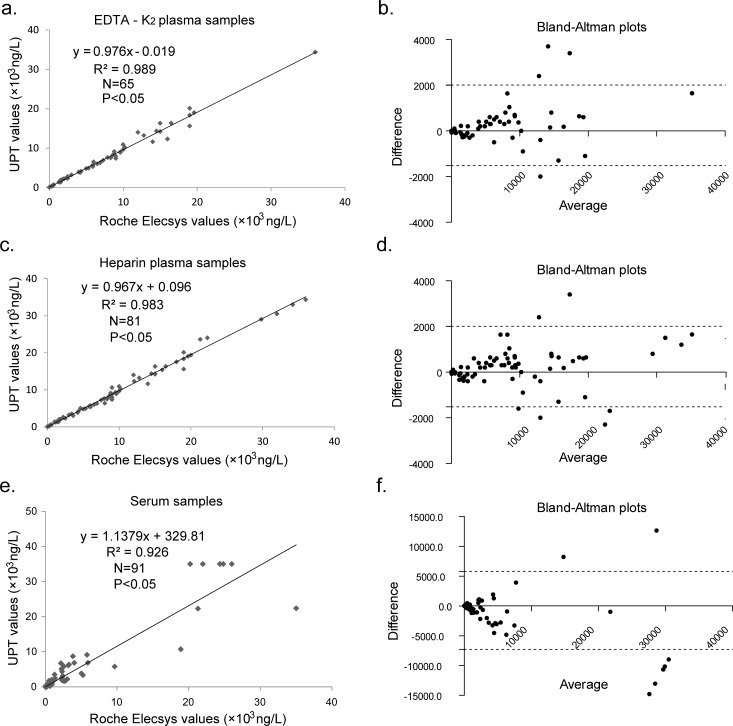
Correlation between the UPT-LF and the Roche Elecsys assay. EDTA-K2 (a) and heparin (c) anticoagulated plasma and serum samples (e) were used to compare the UPT-LF assay and the Roche Elecsys assay, and Bland–Altman plots for these results are also presented (b, d, f), respectively.

To confirm the value of the UPT-LF assay for clinical application, we compared the results from the UPT-LF assay with the Roche Elecsys assay using 65 EDTA-K2 anticoagulated, 81 heparin-anticoagulated plasma and 91 serum samples. The unary linear regression equation for these samples are Y_UPT_ = 0.976X_Elecsys_ − 0.019 (R = 0.995, 95% confidence interval (CI) of the rate and intercept were 0.951–1.002 and −0.346–0.308, *p*<0.05), Y_UPT_ = 0.967X_Elecsys_+0.096 (R = 0.992, 95% CI of the rate and intercept were 0.939–0.995 and −0.272–0.465, *p*<0.05) and Y_UPT_ = 1.138X_Elecsys_+329.81 (R = 0.926, *p*<0.05) respectively (Fig 4). Although the results of the Bland–Altman bias test showed a bias of −1.36, −1.58, and −11.93% between the two methods indicating the good consistency (Fig 4B, 4D and 4F and Table 1), the results of the paired t-test showed a statistically significant difference between the two systems, especially when using EDTA-K2 plasma (*p* = 0.037) and serum (*p* = 0.032) (Table 1). Because of the differences between the two systems, patients should always adhere to one method when they require consecutive tests.

**Table 1:** The correlation between the UPT-LF and the Roche Elecsys assay.

Analytical method	Parameter	Samples		
		EDTA-K2 plasma	Heparin plasma	Serum
**Paired T-test (Two-tailed)**	**P value**	0.037	0.055	0.032
	**Mean of differences**	247.7	190.1	-761.1
	**95% confidence interval**	15.20 ~ 480.2	-4.04 ~ 384.2	-1457 ~ -65.20
	**R squared**	0.07	0.05	0.05
**Bland-Altman plots**	**Bias**	-1.36%	-1.58%	-11.93%
	**SD of bias**	19.25%	20.66%	62.00%
	**95% Limits of Agreement**	-39.09% ~ 36.36%	-42.08% ~ 38.91%	-133.45% ~ 109.59%

### Parallel testing of different sample types using the UPT-LF assay

**Fig 5:**
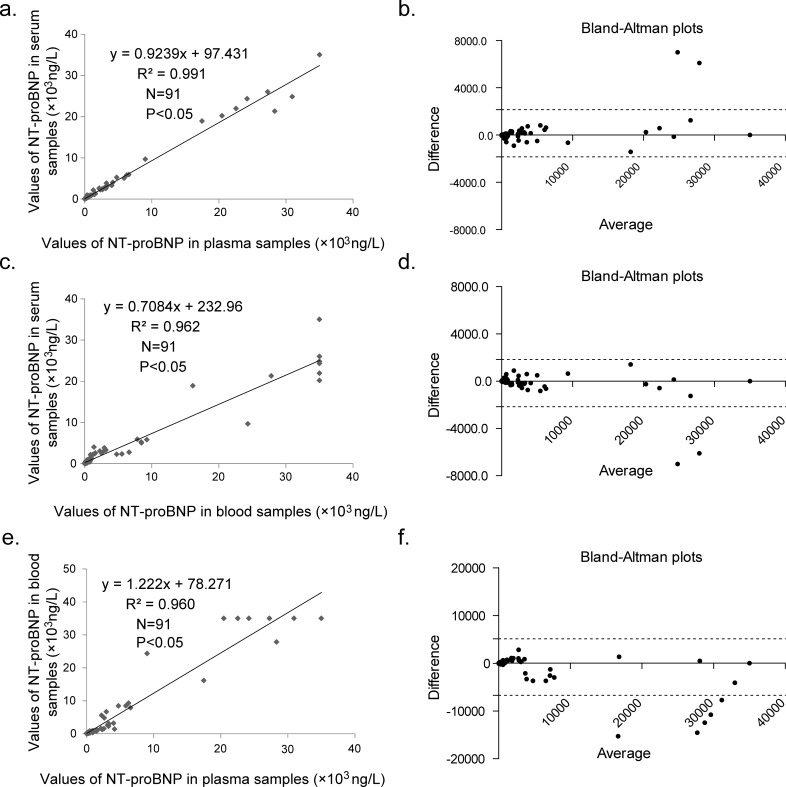
Correlation analysis of three types of samples on the UPT system. Correlation analysis between serum and plasma (a), whole blood and serum (c), whole blood and plasma (e) using the UPT system, and Bland–Altman plots for these results (b, d, f) are presented.

Three types of blood samples were collected (whole blood, plasma, serum) from 91 patients and parallel tests using the UPT-LF assay were performed. As shown in Fig 5A, the correlation analysis showed that the three types of specimens were highly correlated with each other. The correlation coefficient between the serum and plasma samples was 0.99 (*p* = 0.000)(Fig 5A), while those between the whole blood and serum or plasma samples were all above 0.96 (Fig 5C and 5E). As shown in Fig 5B, 5D and 5F and Table 2, the bias was 11.54, −8.02 and −17.99%, respectively. The results of a paired t-test showed no significant difference between serum and plasma samples (*p* = 0.158), while there are statistically significant differences between blood and plasma (*p* = 0.013) or serum (*p* = 0.006) (Table 2). These results indicate that the alternate use of plasma and serum except blood is feasible for consecutive testing when using the UPT-LF assay.

**Table 2:** Paired T-test (Two-tailed) and Bland–Altman plots for three type of samples analyzed using the UPT-LF assay.

Analytical method	Parameter	Samples		
		Plasma vs blood	Serum vs plasma	Serum vs blood
**Paired T-test (Two-tailed)**	**P value**	0.013	0.158	0.006
	**Mean of differences**	-806.4	152.2	958.6
	**95% confidence interval**	-1437 ~ -175.9	-60.62 ~ 365.1	276.8 ~ 1640
	**R squared**	0.07	0.02	0.08
**Bland-Altman plots**	**Bias**	11.54%	-8.02%	-17.99%
	**SD of bias**	73.81%	44.81%	79.37%
	**95% Limits of Agreement**	-133.12% ~ 156.21%	-95.84% ~ 79.80%	-173.56% ~ 137.58%

#### The distribution of blood plasma NT–proBNP in healthy individuals according to age and sex

**Fig 6:**
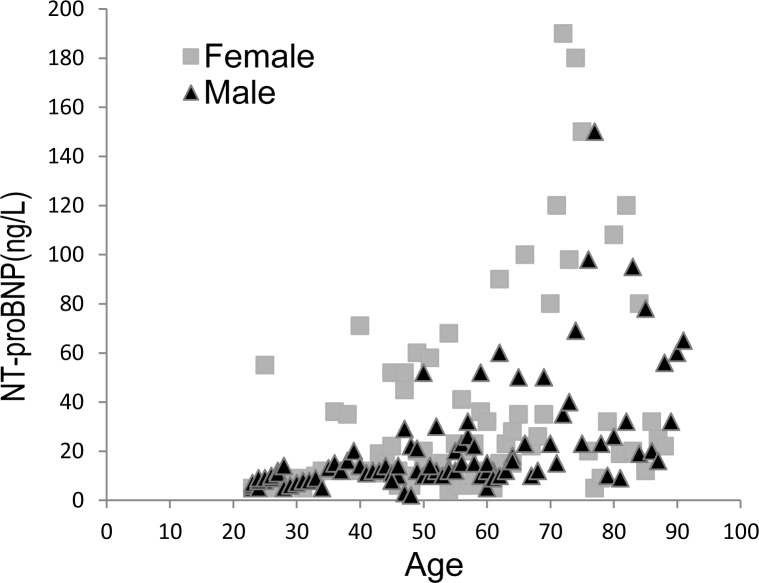
Distribution of blood plasma NT-proBNP (with EDTA-K2 anticoagulant) in healthy individuals is influenced by both age and sex. The regression formula in females: Y = 2.96X−92.35 (R^2^ = 0.805, *p* = 0.001, N = 100); males: Y = 0.76X-10.08 (R^2^ = 0.302, *p* = 0.001, N = 100); total: Y = 1.62X−32.37 (R^2^ = 0.496, *p* = 0.001, N = 200).

Many studies have shown that heart failure is an age-related disease and NT-proBNP levels increase with age [34, 35]. With the aim of demonstrating the superior clinical performance of the UPT-LF assay, we detected the concentration of NT-proBNP in EDTA-K2-anticoagulated plasma from 200 healthy people with an age of 22–91 (median 53) years using the UPT-LF assay. As shown in Fig 6, the level of plasma NT-proBNP increased with age and had a relationship with sex, especially in females, where it was increased (z = 2.864, *p* < 0.01).

### Conclusions

This is the first time a UPT-LF assay for the quantitative detection of NT-proBNP has been reported. Our results demonstrate that it has a high sensitivity, a wide linear range, and excellent repeatability and stability. Importantly, the results are consistent with those obtained using a Roche Elecsys assay while using a shorter test time of less than 20 minutes. Different blood anticoagulants and sample types (serum and plasma) have little effect on the detection results of the UPT-LF assay, demonstrating its feasibility to meet clinical requirements. The UPT biosensor used in the study is a small and portable instrument with a built-in battery, all of which satisfy the requirements of POCT detection. In summary, the UPT-LF assay is a candidate POCT method for the detection of NT-proBNP, especially for acute heart failure.

## Materials and Methods

### Ethics statement

All experiments were performed in accordance with the Guidelines for the Welfare and Ethics of Laboratory Animals of China. All experimental protocols were approved by the Ethics Committee of the General Hospital of Chinese People’s Armed Police Forces. Written informed consent was obtained from all patients, and all of the patient records and information were anonymized and de-identified prior to analysis. The samples in this study were clinical samples remaining after all other clinical testing, and could essentially be considered biological waste.

### Materials

UCP (NaYF4:Yb^3+^, Er^3+^) nanoparticles, with excitation and emission spectrum peaks of 980 nm and 541.5 nm respectively, was provided by Dr. Yan Zheng from Shanghai Kerune Phosphor Technology Co., Ltd. (Shanghai, China). Nitrocellulose membrane (SHE 1350225) and glass fiber (GFCP20300) were obtained from Millipore Corp. (Bedford, MA, USA). Lamination Card and absorbent papers (Nos 470 and 903) were purchased from Schleicher & Schuell, Inc. (Keene, NH, USA). Plastic cartridges for strips were designed by our group and produced by Shenzhen Jincanhua Industry Co. (Shenzhen, China). UPT biosensor, a portable instrument with internal battery and strip scanner, was provided by Shanghai Institute of Optics and Fine Mechanics, Chinese Academy of Sciences (Shanghai, China). The antibodies for fabrication of UPT-LF strip were all prepared by our laboratory and screened by ELISA method.

### Sample collection

Blood samples were collected from 200 healthy people enrolled in the General Hospital of Chinese People’s Armed Police Forces (age 22–91 years, median age 53 years, 100 male subjects and 100 female subjects) and 197 patients with heart failure (age 40–85 years, median age 57 years) from March of 2013 to March of 2014. Any subjects with jaundice, hemolysis and chyle that may influence the results for other clinical testing of patient were excluded during the sample collection process. In this study, we focus on POCT testing for acute heart failure, therefore based on the medical history, a physical examination, a laboratory examination and an electrocardiogram of the patients, we excluded those individuals with hypertension, congestive heart failure, coronary heart disease, diabetes and kidney disease, who have or may have chronic heart failure. We also excluded female patients who were taking birth control pills or oral estrogen because the estrogen in these medicines may influence the NT-proBNP level.

### Establishment of UPT-LF assay

For conjugate pad preparation, mouse anti-human NT-proBNP IgG 1 with a final concentration of 0.1 mg/ml were mixed with 1mg/ml of UCP particles for label [36]. After labeled, 1 mg/ml of UCP-NT-proBNP IgG 1 conjugates were pour into the glass fiber (10 mm wide) and then dried at 37°C for 1h. 2 mg/ml of goat anti-mouse IgG polyclonal antibody and mouse anti-human NT-proBNP IgG 2 prepared by phosphate buffer were dispensed on the nitrocellulose membrane as T and C line of analytical membrane using Imagine Isoflow Reagent Dispenser (Imagene Technology Inc., USA), and then dried at 37°C for 1h. For UPT-LF strip fabrication, sample pad (glass fiber), conjugate pad, analytical membrane, absorbent papers were attached to lamination card successively. For detection, samples were mixed with sample treating buffer (0.03 mol/L phosphate buffer containing 2% BSA, 0.5% NP-40 and 0.25 mol/L NaCl) at a ratio of 1:9, and then 100 μL mixtures were applied directly to the strip. After 15 min, the strip was scanned by the strip reader of UPT biosensor. The ratio between signal on T and C line, namely T/C ratio, was employed as the detection results [31, 32].

### Sensitivity and linearity assessment

The linear scope for quantitation was determined following the America CLSI standards. Standard samples were diluted from 100%–10%(a serial of 10 concentrations) using 0.01M phosphate buffered saline (pH 7.4), the 10 μL prepared sample were mixed with sample-treating buffer at a ratio of 1:9 and detected using the UPT-LF assay. The linear regression analysis was performed according to the average of the measured and theoretical values.

An NT- proBNP standard with a concentration of 35000 ng/L was diluted 2, 4, 8, 16, 32, 64, 128, 256, and 512-fold, and then the 10 specimens were analyzed for calculation of the CV. Each test was repeated 10 times.

### Performance evaluation of the UPT-LF assay for detection of NT-proBNP in clinical samples

To evaluate the effect of sample storage temperature and time on the results of the UPT-LF assay, clinical samples with NT-proBNP concentrations higher than 1,000 ng/L, as detected by the Roche Elecsys assay were selected and mixed together. The concentration of NT-proBNP was reexamined using both the Roche Elecsys assay the UPT-LF assay (NT-proBNP 6523 ng/L) and then the sample was dispensed into several tubes, which were placed at room temperature for 3 days, 4°C for 7 days, −20°C for 30 days, and three repeated freeze-thaw cycles (thawed at room temperature). When this was complete, each sample was applied to the UPT-LF strip after mixed with sample treating buffer, and these results were compared with the original data.

To evaluate the effect of anticoagulants on the UPT-LF assay [33], we detected the concentrations of NT-proBNP in EDTA-K2- and heparin-treated specimens from 40 patients with heart failure, twice, using a UPT-LF assay, and compared the differences between them.

To analyze the correlation between the UPT-LF and Elecsys assays, 65 specimens of EDTA-K2-anticoagulated plasma, 81 specimens of heparin-anticoagulated plasma and 91 serum samples were analyzed twice, using the two methods. The average values were analyzed for the correlation and deviation between them.

To evaluate the effects of different sample types on the UPT-LF assay, serum, plasma, and whole blood from 91 patients were analyzed twice using the method. The average values were analyzed for the correlation and deviation between them.

To explore the distribution of blood plasma NT–proBNP in healthy individuals according to age and sex, plasma samples from 200 healthy people stored in EDTA-K2 anticoagulant, were evaluated using the UPT-LF assay, as described above.

### Statistical analysis

SPSS 16.0 and Medcalc 8.1.0 software packages were used for data processing. Monadic linear regression analysis was used to compare the two anticoagulants and the results of the two methods, in which the correlation (r) ≥0.99 means a significant correlation. Paired t-tests and Bland–Altman plots were used to evaluate the consistency between the two methods. Passing–Bablok regression was used to analyze the NT-proBNP levels among different sex/age groups. The linear regression was analyzed using the Cusum method, *p* <0.05 indicated that the regression had statistical significance.
